# Causes and consequences of cerebral small vessel disease. The RUN DMC study: a prospective cohort study. Study rationale and protocol

**DOI:** 10.1186/1471-2377-11-29

**Published:** 2011-02-28

**Authors:** Anouk GW van Norden, Karlijn F de Laat, Rob AR Gons, Inge WM van Uden, Ewoud J van Dijk, Lucas JB van Oudheusden, Rianne AJ Esselink, Bastiaan R Bloem, Baziel GM van Engelen, Machiel J Zwarts, Indira Tendolkar, Marcel G Olde-Rikkert, Maureen J van der Vlugt, Marcel P Zwiers, David G Norris, Frank-Erik de Leeuw

**Affiliations:** 1 Donders Institute for Brain, Cognition and Behaviour, Centre for Neuroscience, Department of Neurology, Radboud University Nijmegen Medical Centre, PO Box 9101, 6500 HB Nijmegen, The Netherlands; 2 Donders Institute for Brain, Cognition and Behaviour, Centre for Neuroscience, Department of Psychiatry, Radboud University Nijmegen Medical Centre, Nijmegen, The Netherlands; 3 Donders Institute for Brain, Cognition and Behaviour, Centre for Neuroscience, Department of Geriatrics, Radboud University Nijmegen Medical Centre, Nijmegen, The Netherlands; 4 Radboud University Nijmegen Medical Centre, Department of Cardiology, PO Box 9101, 6500 HB Nijmegen, The Netherlands; 5 Donders Institute for Brain, Cognition and Behaviour, Centre for Cognitive Neuroimaging, Radboud University Nijmegen, PO Box 9101, 6500 HB Nijmegen, The Netherlands

## Abstract

**Background:**

Cerebral small vessel disease (SVD) is a frequent finding on CT and MRI scans of elderly people and is related to vascular risk factors and cognitive and motor impairment, ultimately leading to dementia or parkinsonism in some. In general, the relations are weak, and not all subjects with SVD become demented or get parkinsonism. This might be explained by the diversity of underlying pathology of both white matter lesions (WML) and the normal appearing white matter (NAWM). Both cannot be properly appreciated with conventional MRI. Diffusion tensor imaging (DTI) provides alternative information on microstructural white matter integrity. The association between SVD, its microstructural integrity, and incident dementia and parkinsonism has never been investigated.

**Methods/Design:**

The RUN DMC study is a prospective cohort study on the risk factors and cognitive and motor consequences of brain changes among 503 non-demented elderly, aged between 50-85 years, with cerebral SVD. First follow up is being prepared for July 2011. Participants alive will be included and invited to the research centre to undergo a structured questionnaire on demographics and vascular risk factors, and a cognitive, and motor, assessment, followed by a MRI protocol including conventional MRI, DTI and resting state fMRI.

**Discussion:**

The follow up of the RUN DMC study has the potential to further unravel the causes and possibly better predict the consequences of changes in white matter integrity in elderly with SVD by using relatively new imaging techniques. When proven, these changes might function as a surrogate endpoint for cognitive and motor function in future therapeutic trials. Our data could furthermore provide a better understanding of the pathophysiology of cognitive and motor disturbances in elderly with SVD. The execution and completion of the follow up of our study might ultimately unravel the role of SVD on the microstructural integrity of the white matter in the transition from "normal" aging to cognitive and motor decline and impairment and eventually to incident dementia and parkinsonism.

## Background

Cerebral small vessel disease (SVD) includes white matter lesions (WML) and lacunar infarcts and is a frequent finding on computer tomography (CT) and magnetic resonance imaging (MRI) scans of elderly people [1]. It is associated with vascular risk factors, such as hypertension, atherosclerosis, diabetes mellitus and atrial fibrillation [2-4]. In cerebral SVD symptoms are due to either complete (lacunar syndromes) or incomplete infarction (WML) of subcortical structures leading to accompanying complaints including the lacunar syndromes, cognitive, motor (gait) and/or mood disturbances [5]. The prevalence of WML and lacunar infarcts varies considerably across studies from 5-95% and 8-28% respectively, depending on the population studied and the imaging technique used [1,6]. There is evidence of an increased risk of cognitive decline, dementia, gait and balance disturbances and parkinsonism among individuals with SVD, although prospective studies are scarce [7-10].

However, individuals with a virtually identical WML burden on conventional FLuid Attenuated Inversion Recovery (FLAIR) imaging present with a wide variance in cognitive and motor performance ranging from no complaints at all to subjective cognitive complaints and mild parkinsonian signs to dementia and parkinsonism. Apparently there are other factors that determine whether identical appearing WML on FLAIR lead to for example cognitive or motor decline in one person, while leaving others unaffected.

One of the other factors could be the presence the coexisting manifestations of cerebral SVD on conventional MRI such as lacunar infarcts and cerebral microbleeds which might influence the cognitive and motor performance [11].

As identical appearing WML on conventional MRI are actually histopathologically heterogeneous [12], it could be that only the WML with a high loss of microstructural integrity are related to cognitive and motor impairment. It is also important to realize that only a small proportion of the white matter (usually less than a few percent) is affected by SVD, even among individuals with severe SVD [13]. As conventional MRI is not sensitive to early loss of microstructural integrity in the normal appearing white matter (NAWM), possible changes in this largest part of the white matter cannot be assessed [14,15]. These limitations of conventional MRI can potentially be overcome with the use of Diffusion Tensor Imaging (DTI) which allows us to assess the microstructural integrity of the whole white matter [16]. DTI, amongst others, provides two parameters; mean diffusivity (MD), a measure of the magnitude of diffusion of water in the white matter, and fractional anisotropy (FA), which provides information about the directionality of water diffusion. Damage to the white matter is supposedly accompanied roughly by an increase in MD and a decrease in FA [17].

Another explanation for the clinical diversity due to WML could be the efficiency of compensation mechanisms that prevent further cognitive and motor (gait) deterioration. Support for the existence of compensatory mechanisms comes from a study among young carriers of a pre-senilin mutation (at risk for genetically determined Alzheimers' disease (AD), but still without cognitive impairment) who showed altered functional connectivity (assessed with fMRI) compared with controls [18]. With innovative resting state fMRI techniques the strength of functional connectivity between brain regions can be investigated [19]. In that way it might be that these compensation mechanisms also play a role in the variety of clinical presentation of individuals with SVD.

In the RUN DMC (Radboud University Nijmegen Diffusion tensor and Magnetic resonance imaging Cohort) study we prospectively investigate the effect of SVD on the transition from non-demented, independently living elderly people with cerebral SVD between 50 and 85 years towards cognitive and motor (gait) decline, and ultimately dementia and parkinsonism in a population with cerebral SVD. The primary objective of the RUN DMC study is to prospectively investigate the risk factors for and cognitive and motor (gait) consequences of longitudinal functional and structural changes in the integrity of the cerebral white matter as assessed by DTI, resting state fMRI and conventional structural MRI. To the best of our knowledge there are no other prospective cohort studies investigating the development of incident dementia and parkinsonism using these novel imaging techniques. Here we describe the study design and protocol of the RUN DMC study.

## Methods/Design

### Study population

Cerebral SVD is characterized on neuroimaging by either WML or lacunar infarcts. Symptoms of SVD include acute symptoms, such as transient ischemic attack (TIA) or lacunar syndromes, but also subacute manifestations such as cognitive and motor (gait) disturbances [5]. As the onset of cerebral SVD is often insidious, clinically heterogeneous, and typically with mild symptoms, it has been suggested that the selection of subjects with cerebral SVD in clinical studies should be based on the more consistent brain imaging features [20].

Accordingly, in 2006, consecutive individuals referred to the Department of Neurology between October 2002 and November 2006, were selected for possible participation. Inclusion criteria were: (a) age between 50 and 85 years; (b) cerebral SVD on neuroimaging (WML and/or lacunar infarcts). Subsequently, the above mentioned acute and subacute clinical symptoms of SVD were assessed by standardized structured assessments (a questionnaire for TIA and stroke [21]; for cognition the Cognitive Failures Questionnaire [22]; for gait the Falls Questionnaire [23] and the Freezing of Gait Questionnaire [24]) Subjects who were eligible because of a lacunar syndrome were included only > 6 months after the event to avoid acute effects on the outcomes.

To be able to detect incident dementia and parkinsonism we applied the following exclusion criteria: (a) presence of dementia [25] and (b) parkinson(-ism)[26,27]. In addition patients with (c)intracranial hemorrhage; (d) life expectancy of less than six months; (e) intracranial space occupying lesion; (f) (psychiatric) disease interfering with cognitive testing or follow-up; (g) recent or current use of acetylcholine-esterase inhibitors, neuroleptic agents, L-dopa or dopa-a(nta)gonists; (h) non-SVD related WML (e.g. multiple sclerosis); (i) prominent visual or hearing impairment; (j) language barrier; (k) MRI contraindications or known claustrophobia were excluded.

All participants signed an informed consent form. The Medical Review Ethics Committee region Arnhem-Nijmegen approved the study.

### Follow-up

After 5 and 10 years all participants alive will be contacted for the prospective assessment of possible outcome events. This evaluation is currently being prepared for July 2011.

Between 2006 and 2011 we contacted all participants every year by letter for an update on their address information and telephone number and for their survival status.

In 2011 all participants alive will be invited by letter and subsequently contacted by telephone to visit our research centre. During their visit to the research centre a cognitive, gait, balance and parkinsonian signs assessment, a structured interview, physical examination, neurological examination, and an extensive MRI protocol, an electrocardiogram and an ultrasonography of the carotid arteries will be performed. All tests will be performed by the same two trained neurology residents and all MRI scans will take place on the same scanner.

### Outcome events

Primary measures of outcome of the study are incident dementia and parkinsonism according to international diagnostic criteria[25,27], as well as all-cause mortality and death from all vascular causes, non-fatal stroke, and non-fatal myocardial infarction.

Secondary outcome measures are defined as change from baseline examination in cognitive function, gait and balance and parkinsonian signs.

Incident outcome events are to be identified by three different approaches.

1. During the follow-up a structured questionnaire on the possible occurrence on these outcome events is administered to each participant. When an incident event is suspected the treating physician will be contacted for the most recent information on that particular outcome event.

2. When a participant died before follow-up, the general practitioner will be contacted for the most recent information on the cause of death and presence of primary outcome events. In case of presence of primary outcome events the treating physician will be contacted for the most recent information available.

3. When during follow-up assessment participants' test results are suggestive for incident dementia or parkinsonism, subjects will be referred to our outpatient clinic. In case the diagnosis is established according to the international criteria, this will be considered an incident case.

All outcome events will be adjudicated independently by two specialised physicians, if the two classifications differ, the outcome event will be discussed and consensus will be made.

### Assessment of cognitive and motor outcomes

Two trained residents in neurology will administer the complete outcome assessment.

#### Cognitive assessment

We will use an extensive neuropsychological test battery that encompasses items from other large scale epidemiological studies that cover virtually all cognitive domains [10,28]. A measurement of global cognitive function will be assessed by the Mini Mental State Examination (MMSE) [29]. The verbal memory function will be assessed by the three-trial version of the Rey Auditory Verbal Learning Test (RAVLT), a test used to evaluate the ability to acquire and retain new verbal information [30]. Visuospatial memory will be administered by the Rey's Complex Figure Test (RCFT), that consists of three subtasks: the copy trial, the immediate recall trial, within 3 minutes and the delayed recall trial, after 30 minutes [31]. To evaluate speed of mental processes four tests will be used; the Stroop test (three subtasks) [32], the Paper and Pencil Memory Scanning Task (four subtasks) [33], the Symbol-Digit Substitution Task, which is a modified version of the Symbol Digit Modalities Test [34] and a verbal fluency task in which as many animals as possible have to be named within 60 seconds, followed by as many professions within 60 seconds. To evaluate attention, the verbal series attention test (VSAT) will be used [35]. To register subjective cognitive failures we will administer the Cognitive failures questionnaire (CFQ) [22]. The tests will be carried out in quiet rooms and a stopwatch will be used in timed tests.

#### Assessment of gait, balance and parkinsonian signs

All participants will perform a tandem walk by walking ten steps heel to toe (registering: intact, one side step, more side steps, impossible). A quantitative gait analysis will be performed with a 5.6-meter long, 0.89-meter wide electronic walkway (GAITRite^® ^MAP/CIR Inc., Havertown, PA) with sensor pads (12.7 mm apart from each other) connected to a computer. This system has strong concurrent validity and test-retest reliability, also in older people [36]. The participants walk twice at self-selected gait speed on low-heeled shoes. They start two meters before the carpet and walk until two meters behind it in order to measure steady-state walking.

We will use a widely used modified version of the original Tinetti test with 17 items: 9 for body balance (score 0-16) and 8 for gait (score 0-12), with a maximum score of 28 [37]. It grades balance while sitting, standing with eyes open and closed, nudging and turning, gait initiation, stride length and width and symmetry. Functional mobility will be classified by using the widely-used TUG-test which is a timed test during which the participant is asked to rise from a standard armchair, walk 3 m, turn, walk back and sit down again [38]. Each participant will perform the test three times. To evaluate parkinsonian signs we apply the Unified Parkinson's Disease Rating Scale (UPDRS), the motor score [39]. Finally disease severity will be assessed with the Hoenhn and Yahr stage assessing [40]. For the evaluation of gait and balance we will also administer the Freezing of gait questionnaire (FOG), a questionnaire consisting of 16 items regarding gait and falls and the Falls questionnaire [23,24].

#### Assessment of activities of daily living

As a measure of disability the Barthel Index will be used [41]. The activities of daily living will be assessed by the instrumental activities of daily living questionnaire [42].

#### Structured interview

##### Demographics and life style

Standardized questionnaires on demographics, education (classified using 7 categories, 1 being less than primary school and 7 reflecting an academic degree)[43], marital status, living conditions, and life style habits (alcohol consumption, smoking, exercise) will be administered. Alcohol consumption is defined as units per day and the age at which alcohol consumption had started (and if stopped) was noted. Cigarette smoking behaviour is defined as the number of pack-years, calculated as the number of packs of cigarettes smoked per day multiplied by the number of years a participant had smoked. Exercise is expressed in the metabolic equivalent value (MET) according to accepted standards, where 1 MET is proportional to the energy expended while sitting quietly [44].

##### Vascular risk factors and cardiovascular disease

With the aid a of structured, standardized questionnaire each participant will be asked for a history of: hypertension, diabetes mellitus, atrial fibrillation, TIA, stroke, myocardial infarction, coronary artery bypass graft, per-cutaneous transluminal coronary angiography, aortic prothesis, vascular prothesis, carotid endartectomy [2-4,21] and migraine [45] The presence of a family history of myocardial infarction, cerebrovascular disease and diabetes mellitus in next of kin will be recorded.

##### Current medication

Current medication use will be noted and classified according to the Anatomical therapeutic chemical (ATC) classification system. (World Health Organization, WHO Collaborating Centre for drug statistics and methodology, http://www.whocc.no/atcddd/)

### Assessment of other variables

#### Depressive symptoms

A standardized structured questionnaire used in previous large scale epidemiological studies will be used to assess for the history of depressive symptoms; normal reactions to stressful events or normal grief will carefully be excluded [46]. In case of a depressive episode, age of onset, the medical advice and medication use will be registered. We defined 'depression' as those depressive episodes that have required attention of a general practitioner, psychologist, or psychiatrist. This definition includes minor depression, as well as more severe depression syndromes such as major depression and bipolar depression [46].

In addition participants will be screened for depressive symptoms by means of the Mini International Neuropsychiatric Interview (MINI), part A, which is a short diagnostic structured interview based on the DSM IV [47]. Additionally, presence of actual depressive symptoms will be assessed by two self report questionnaires, the Center of Epidemiologic Studies Depression Scale (CES-D) [48] and the Hospital Anxiety and Depression Scale (HADS) [49].

#### Additional Self-report questionnaires

For the assessment of sleep disorders we will use the SCOPA-Sleep scale [50] and for fatigue the Checklist on Individual Strength (CIS20R) [51]. The overall health status (quality of life) will be assessed with the Short Form 36 (SF-36) [52,53].

#### Physical Examination

Height and weight will be measured without shoes in light clothing. The body mass index (BMI) is calculated as weight divided by height (in meters) squared. The maximal waist circumference will be measured without shirt, in standing position, between the lowest rib and the iliac crest, at the end of normal expiration [54]. Blood pressure and pulse rate will be measured in triplicate in supine position after 5 minutes rest. Subsequently one measurement is performed after 1 minute in upright position [3].

#### Neurological examination

##### Primary reflexes

The presence of the glabella, snout and grasp reflex, the applause sign [55] and the plantar response will be registered.

##### Muscle strength

The strength of the biceps, hand grip, iliopsoas, quadriceps and foot extensor muscles on both sides will be measured by the medical research council scale (MRC) and by a dynamometer. (Citec^® ^hand-held dynamometer) [56].

*Sensory system *will be assessed by a quantitative measurement by vibration tuning fork (Rydel-Seiffer^®^) on both first toes and both medial malleolus, also registering ankle oedema and ankle jerks.

### Ancillary Investigation

#### MRI protocol

MRI scanning will be performed on a 1.5-Tesla Magnetom scanner (Siemens, Erlangen, Germany). The scanning protocol includes whole brain 3 D T1 magnetization-prepared rapid gradient-echo (MPRAGE) sequence (TR/TE/TI 2250/3.68/850ms; flip angle 15°; voxel size 1.0 × 1.0 × 1.0 mm); FLAIR pulse sequences (time repetition [TR]TE/TI 9000/84/2200 ms; voxel size 1.0 × 1.2 × 6.0 mm (including slice gap of 1 mm); transversal T2* weighted gradient echo sequence (TR/TE 800/26 ms; voxel size 1.3 × 1.0 × 6.0 mm (including slice gap of 1.0 mm); DTI (TR/TE 10100/93ms; voxel size 2.5 × 2.5 × 2.5 mm; 4 unweighted scans, 30 diffusion weighted scans, with non co-linear orientation of the diffusion-weighting gradient, and b value 900 s/mm^2^) and resting state imaging using a gradient echo EPI (TR/TE 2400/40ms; voxel size 3.5 × 3.5 × 4.4 mm (including slice gap of 0.4 mm)). During resting state, subjects will be told not to concentrate on any particular subject, but just to relax with their eyes closed. The complete scanning protocol takes 31 minutes.

##### White matter lesions

All images will be evaluated without prior notice of any clinical parameter. WML are defined as hyperintense lesions on FLAIR MRI without corresponding cerebrospinal fluid like hypo-intense lesions on the T1 weighted image. Gliosis surrounding lacunar and territorial infarcts is not considered to be WML [57]. Total WML volume is calculated by an in-house developed, validated technique.

##### Brain volumetry

Normalization parameters to the ICBM152 linear template (as provided with SPM5; Wellcome Department of Cognitive Neurology, University College London, UK) and gray and

white matter tissue and cerebrospinal fluid probability maps is computed by using SPM5 unified segmentation routines on the T1 MPRAGE images [58]. Total grey and white matter volumes are calculated by summing all voxel volumes that have a p > 0.5 for belonging to the tissue class. Total brain volume is taken as the sum of total grey- and total white matter volume. Co-registration parameters of the FLAIR image to the T1 image are computed (SPM5 mutual information co-registration) and used to bring both the FLAIR and WML segmentation images into the subject's (anatomical) reference frame. Transformed images will visually be checked for co-registration errors. Subsequently, the WML segmentations are resampled to and combined with the white matter maps to yield to a WML map (the intersection of WML and white matter) and NAWM map (the complement of WML in white matter) in the T1 reference space. Total brain volume is taken as the sum of total gray and white matter.

##### Lacunar and territorial infarcts

Lacunar infarcts are defined as hypo-intense areas > 2 mm and ≤ 15 mm on FLAIR and T1, ruling out enlarged perivascular spaces (≤ 2 mm, except around the anterior commissure, where perivascular spaces can be large) and infraputaminal pseudolacunes [57]. Territorial infarcts are defined as hyperintense lesions on FLAIR and hypointense lesions on T1 images >15 mm [57].

##### Microbleeds

Microbleeds are defined as small, homogeneous, round foci of low signal intensity on T2* weighted images of less than 10 mm in diameter [59]. Microbleeds are counted per hemisphere separately. In addition they are classified as cortical/subcortical including the periventricular white matter and deep portions of the centrum semiovale (frontal, parietal, occipital and temporal separately); in the basal ganglia, including caudate nucleus, internal and external capsule, globus pallidus, thalamus and putamen; infratentorial including the cerebellar hemispheres, pons and medulla oblongata [59]. Lesions are not considered to be microbleeds when they are symmetric hypointensities in the globus pallidus, most likely calcifications or iron deposits, flow voids artifacts of the pial blood vessels or hyposignals in T2* inside a lesion compatible with an infarct, likely to be hemorrhagic transformation [59].

##### Diffusion tensor imaging

The diffusion weighted images of each participant are realigned on the unweighted image using mutual information based Matlab (The Mathworks, Inc.) routines from SPM5. Then, the diffusion tensor and its eigenvalues are computed using an SPM5 add-on http://sourceforge.net/projects/spmtools[60]. Unphysical spurious negative eigenvalues of the diffusion tensor were set to zero, after which the tensor derivatives the FA and MD are calculated [61]. The mean unweighted image is used to compute the co-registration parameters to the anatomical T1 image (SPM5 mutual information co-registration), which are then applied to all diffusion weighted images and results. All images are visually checked for motion artefacts and coregistration errors.

### Electrocardiogram

An electrocardiogram (ECG) will be performed and evaluated by a standardized assessment by an experienced cardiologist, registering frequency, cardiac rhythm, cardiac ectopias, cardiac axis, conduction time over the PQ, QRS and QTC intervals, conduction disturbances, left ventricle hypertrophy, pathologic Q's, infarction, repolarisation disturbances and acute ischemia. A final diagnosis is defined as normal, abnormal without clinical significance, abnormal with clinical consequences or pathologic ECG with immediate consultation of a cardiologist when necessary.

### Ultrasonography of the carotid arteries

All ultrasound measurements will be performed by three experienced and specific trained clinical neurophysiology technicians. A carotid ultrasound assessment at which the intima media thickness (IMT) is measured in the distal left and right carotis communis, near the bulbus, will be performed. All measurements will be performed using a phased array real-time scanner (Philips i-u22, The Netherlands) with a 17-5 MHz broadband linear transducer. Two-dimensional ultrasound imaging of the carotid artery will be performed to measure the IMT. The IMT will be automatically measured by QLab^® ^qualification software (V. 4.2.1.). An edge detection algorithm identified the lumen/intima and the media/adventitia interfaces within a region of interest over a 10 mm long segment and calculated the average thickness [62].

The same cognitive, motor, gait and balance assessment, structured interview and assessment of other variables and the same ancillary investigation were performed at baseline in 2006.

### Statistical analysis

#### Sample size calculation

Based on the literature we expect about 60 incident dementia cases during the five year follow up (absolute risk 4-5%/year), as about half of our study population has a relatively high degree of WML [63]. We expect that each SD increase in MD increases this absolute risk of dementia by 2% per year. To detect this increased risk with a high probability of 90% at the 5% significance level we will need 380 participants at the end of the follow up, so therefore we included 500 participants at baseline and hope to end up with 400 participants at follow-up protocol (taking into account an expected loss to follow up of about 20%).

#### Analysis of primary outcome measures

We will analyze mean baseline MD and FA and change in MD and FA on follow up imaging in relation to incident dementia and parkinsonism by Cox proportional hazard models adjusted for age, sex, education, depressive symptoms, total brain volume, white matter lesion volume and lacunar infracts, where appropriate.

## Discussion

The RUN DMC study is a large prospective cohort study on causes and consequences of structural and functional changes in the integrity of the cerebral white matter (in both the WML and the NAWM) as assessed by conventional MRI as well as new techniques, such as DTI and resting state fMRI, among elderly with cerebral SVD, starting to include participants for the follow-up protocol in July 2011.

Numerous studies have shown that WML observed on conventional MRI are related to vascular risk factors and have reported associations with cognitive and motor decline and found these relations to be rather weak [3,4,8,10,46]. To the best of our knowledge there are no prospective cohort studies on individuals with cerebral SVD investigating the development of incident dementia and parkinsonism in relation to white matter changes assessed by DTI and resting state fMRI.

Strengths of the RUN DMC study include the prospective fashion of the study in which all vascular risk factors, clinical and imaging measures will be followed up after five years, and the large and well-established protocol used to explore demographics, vascular risk factors, and cognitive and motor function. The tests chosen are furthermore widely accepted and have been proven specific and sensitive in this population with structural brain changes.

Another strength is the fact that it is a single centre study. Moreover, the complete study protocol will take place in one research centre with the use of a single scanner and only two investigators performing all investigations.

In conclusion, the RUN DMC study has the potential to further unravel the causes and consequences of changes in white matter integrity in elderly with cerebral SVD by using new imaging techniques, DTI and resting state fMRI. When proven, changes in white matter integrity assessed by these techniques might function as a surrogate endpoint for cognitive and motor function in future therapeutic trials of vascular risk factors in SVD.

The execution and completion of the follow-up of our study will ultimately unravel the role of SVD on the microstructural integrity of the white matter in the transition from "normal" aging to cognitive and motor decline and impairment and eventually to incident dementia and parkinsonism.

## Competing interests

The authors declare that they have no competing interests.

## Authors' contributions

AvN, KdL, FEdL: contribution to conception and design; acquisition of data; involvement in drafting the manuscript; final approval of the version to be published

RG, IvU, LvO: acquisition of data; revising the manuscript critically; final approval of the version to be published

EvD, RE, BB, BvE, MZ, IT, MOR, MvdV, MZ, DN: contribution to conception and design; revising the manuscript critically; final approval of the version to be published

## Pre-publication history

The pre-publication history for this paper can be accessed here:

http://www.biomedcentral.com/1471-2377/11/29/prepub
